# Topical frankincense treatment on relieving high-risk diabetic foot in rats by reducing inflammation and improving microcirculation

**DOI:** 10.3389/fphar.2025.1564236

**Published:** 2025-09-16

**Authors:** Si-Yuan Ma, Xin Yin, Zhao Zhang, Yu-Sang Li, He-Bin Tang

**Affiliations:** ^1^ Lab of Hepatopharmacology and Ethnopharmacology, School of Pharmaceutical Sciences, South-Central Minzu University, Wuhan, China; ^2^ Department of Pathophysiology, School of Basic Medical Sciences, Wuhan University, Wuhan, China; ^3^ Tongcheng Traditional Chinese Medicine Hospital, Tongcheng, Anhui, China

**Keywords:** diabetes, high-risk diabetic foot, frankincense, blood vessel damage, nerveconduction velocity

## Abstract

**Background:**

Frankincense, an ancient aromatic substance known for promoting blood circulation, is widely used in traditional Chinese medicine to treat skin lesions.

**Methods:**

This study aimed to evaluate the efficacy and underlying mechanisms of frankincense oil extract (FOE) in addressing high-risk diabetic foot by improving microvascular circulatory disorders. The vascular repair effects of FOE were assessed in a streptozotocin-induced high-risk diabetic foot animal model. Various evaluations were performed, including infrared thermometry, motor nerve conduction velocity (MNCV) testing, laser Doppler flowmetry (LDF), hematoxylin and eosin (H&E) staining, and immunohistochemical analyses for CD31, TRPV3, β-catenin, MMP-9, and COX-2 expressions.

**Results:**

The results showed that, the femoral arteries, plantar arteries, and microvessels in high-risk diabetic foot rats displayed significant damage, evidenced by reduced blood flow velocity, ruptured vascular intima, and disorganized endothelial fibers. FOE treatment restored temperature and blood flow velocity in the foot and increased MNCV. Furthermore, FOE improved blood circulation, reversed the reduced expression of CD31, enhanced nerve sensitivity by up-regulating TRPV3 expression, and exhibited anti-inflammatory effects by reducing the overexpression of β-catenin, MMP-9, and COX-2.

**Conclusion:**

FOE effectively prevents high-risk diabetic foot by repairing vascular damage, restoring blood circulation, enhancing nerve sensitivity, and suppressing inflammatory cell infiltration. Therefore, these findings highlight the potential clinical value of FOE in managing diabetic complications.

## 1 Introduction

Statistics from the International Diabetes Federation indicate that the global prevalence of diabetes reached approximately 537 million in 2021 and is projected to exceed 783 million by 2045 (Sun et al., 2022). It is estimated that up to 34% of individuals with diabetes may develop diabetic foot ulcers (DFU; Armstrong et al., 2023). High-risk diabetic foot represents the early stage of DFU, during which diabetic patients exhibit high-risk factors such as vascular and nerve lesions, but no visible wounds or infections on the foot (Bus et al., 2016).

The primary pathogenic mechanisms underlying diabetic foot include vasculopathy, neuropathy, and infection (Deng et al., 2023). Hyperglycaemia in diabetes leads to dysfunction of the vascular endothelium, which disrupts microvascular circulation (Shah and Brownlee, 2016). Endothelial dysfunction further impacts a variety of cellular functions and inflammatory factors, such as β-catenin, COX-2, and MMP-9 (Foulquier et al., 2018), along with the cell adhesion molecule CD31 (Szulcek et al., 2016). Additionally, TRPV3 is implicated in diabetic neuropathy (Facer et al., 2007). To manage these symptoms, commonly prescribed drugs in Western medicine include metformin (Chaudhary and Kulkarni, 2024), mecobalamin (Kimber-Trojnar et al., 2022), and voltaren (Derry et al., 2015). However, none of these treatments directly address vascular lesions.

Frankincense, a traditional Chinese medicine, has long been recognized for its ability to activate blood circulation, remove blood stasis, benefit qi, and nourish blood (Commission, 2020). Previous studies have shown that frankincense improves microcirculation and promotes wound healing by inhibiting β-catenin, COX-2, and MMP-9 expression (Yin et al., 2022; Zhang et al., 2025). Based on these properties, our study aimed to explore whether frankincense, known for its circulatory benefits, can be applied to the treatment of high-risk diabetic foot and evaluate its therapeutic efficacy.

## 2 Materials and methods

### 2.1 Materials and instrumentation

Frankincense, the gum resin derived from Boswellia carterii Birdw (originating from the Ethiopia; identified by Prof. Xiaochuan Ye, Hubei University of Chinese Medicine, Wuhan, China), was procured from the Yinpian Factory, Guangzhou Medicine Company (Guangzhou, China). Frankincense (150 g) was soaked in 1 L sesame oil or water for 7 days at room temperature and subsequently incubated at 100 °C for 2 h. The oil extract of Frankincense was filtered and collected. The Frankincense oil extract [FOE; the extracts of frankincense were detected by GC-MS method as described in Supplementary Figure S1; three components (α-pinene, 0.072 mg/mL; linalool, 0.0593 mg/mL; and 1-octanol, 0.359 mg/mL) were identified in the FOE] used in this study was derived from samples collected in a previous study. The FOE sample (No. 20150313) was stored in the sampling room of Hepatopharmacology and Ethnopharmacology, School of Pharmaceutical Sciences, South-Central Minzu University (Li et al., 2016). Common feed and high-fat diet (HFD) were sourced from Beijing Huafukang Biotechnology Co., Ltd. (Beijing, China). The study utilized the following drugs: streptozotocin (STZ; Shanghai Macklin Biochemical Technology Co., Ltd., China), metformin and mecobalamin (Sigma-Aldrich Trading Co., Ltd., Shanghai, China), voltaren (GSK Consumer Healthcare SARL, Switzerland), and XAV-939 (GlpBio Technology, United States). Antibodies against CD31, TRPV3, β-catenin, MMP-9, and COX-2 were supplied by Abcam Inc. (Cambridge, MA, United Kingdom).

### 2.2 Animal care

Thirty-five male Wistar rats (weighing 180–220 g) were obtained from the Hubei Experimental Animal Research Center. The rats were housed in the SPF-grade animal facility of the School of Pharmaceutical Sciences, South-Central Minzu University, under controlled conditions at 22–25 °C. They had unrestricted access to water and food and underwent acclimatization for 1 week prior to the experiments. This study was approved by the Animal Experiment Ethics Committee of South-Central Minzu University, Wuhan, China (Approval No.: 2019-SCUEC-AEC-001).

### 2.3 Animal model establishment

Initially, 35 rats were randomly assigned to two groups: a control group (5 rats) and a model group (30 rats). After 1 week of adaptive feeding, the model group was fed a HFD (Furman, 2021) for 8 weeks, followed by a 12-h fasting period. The diabetes mellitus model was established by intraperitoneal injection of 30 mg/kg STZ dissolved in citrate buffer (pH 4.5). To confirm hyperglycemia, all rats were orally administered 200 g/L glucose solution at a dose of 2 g/kg, and blood glucose levels were measured from tail-vein samples. Rats in the control group were fed a standard diet and injected intraperitoneally with an equivalent dose of sodium citrate buffer after 8 weeks.

Following 3 weeks of continuous feeding, the successfully modeled diabetic rats were randomly divided into six groups (n = 5 rats per group): the model group, FOE group (0.15 mL/cm^2^, topically applied), metformin group (200 mg/kg by gavage), mecobalamin group (10 mg/kg by gavage), voltaren group (0.15 mL/cm^2^, topically applied), and XAV-939 group (20 mg/kg by intraperitoneal injection). Rats in the control and model groups received equivalent volumes of saline via tube feeding. To induce peripheral circulation disorders, all rats, except those in the control group, were required to walk on ice for 10 min daily for 1 week. Drug treatments were administered during the ice-walking period and continued until week 12.

### 2.4 Behavioral assay

The appearance of the rats throughout the experiment was recorded using a single-lens reflex camera (α300, Sony, Tokyo, Japan). At weeks 1, 8, and 12, all rats were weighed, and their glucose levels were measured using a glucometer (GA-3, Sanuo Biosensor Co., Ltd.). At weeks 1, 8, 10, and 12, the plantar temperatures of the rats were measured using an infrared thermal imaging camera (HIKVISION P10B; Hangzhou, China; Brzezinski et al., 2019). The motor nerve conduction velocity (MNCV) was assessed using a biological signal acquisition and analysis system (BL-420E, Chengdu Taimeng Software Co., Ltd.; Coppey et al., 2001). Additionally, at weeks 1, 10, and 12, the flow velocities of the femoral and plantar arteries were evaluated using laser Doppler flowmetry (LDF; Perimed AB Co.).

### 2.5 Histopathological analysis of vascular and plantar skin tissues

At the conclusion of the experiment, 35 rats were euthanized using carbon dioxide at week 12. Plantar skin and blood vessel tissues were collected, processed, and dehydrated to prepare 3 µm paraffin sections, which were subsequently stained with hematoxylin and eosin (H&E) for histopathological examination.

### 2.6 Scoring of vascular, nerve damage and inflammation in rats

Scoring criteria for vascular damage, nerve damage, and inflammation were established based on histopathological findings and the expression levels of related proteins (Supplementary Tables S1-S3). For vascular damage, the scoring system evaluated parameters including the extent of local thickening of the femoral artery wall, homogeneous transparent structural changes in the arterial media (hyalinosis), endothelial integrity of small blood vessels, the degree of perivascular inflammation and fibrosis, and vascular remodeling near arteriovenous shunts (AV-shunts; Setacci and Ricco, 2011). For nerve damage, assessments included the density of nerve endings and structural alterations in the plantar skin layers (Callaghan et al., 2012). For inflammation scoring, criteria included the degree of inflammatory cell infiltration, changes in local tissue structure, perivascular inflammatory cell infiltration, and the extent of influence from associated factors (Tsalamandris et al., 2019). Each characteristic was scored on a scale from 0 to 3 (0: none, 1: mild, 2: moderate, 3: severe), with a higher total score reflecting greater severity of damage.

### 2.7 Immunohistochemical analysis of the tissues

Paraffin sections were deparaffinized and rehydrated. Endogenous enzymes were inactivated using 3% hydrogen peroxide, followed by antigen retrieval through microwaving in citrate buffer. After blocking with 5% bovine serum albumin, the sections were incubated with the following primary antibodies diluted in PBS: anti-CD31 (1:500), anti-TRPV3 (1:1,000), anti-β-catenin (1:500), anti-COX-2 (1:2000), and anti-MMP-9 (1:1,000). Subsequently, secondary antibodies were applied, and protein expression was visualized under a microscope following staining with 3,3′-diaminobenzidine tetrahydrochloride chromophore. Tissue nuclei were counterstained with hematoxylin. The sections were then dehydrated in a gradient ethanol series and mounted for analysis. As detailed in our previous study, multispectral images were acquired and quantitatively analyzed using the CRi Nuance multispectral imaging system (Cambridge Research and Instruments, Woburn, Massachusett; Yin et al., 2022).

### 2.8 Statistical analysis

Experimental data and graphical representations were analyzed using GraphPad Prism 9.0. Data are expressed as the mean ± SEM. Statistical comparisons between groups were performed using the Tukey test within one-way ANOVA, and *p* < 0.05 was considered statistically significant.

## 3 Results

### 3.1 Diabetes mellitus slowed foot blood flow velocity in rats

**FIGURE 1:**
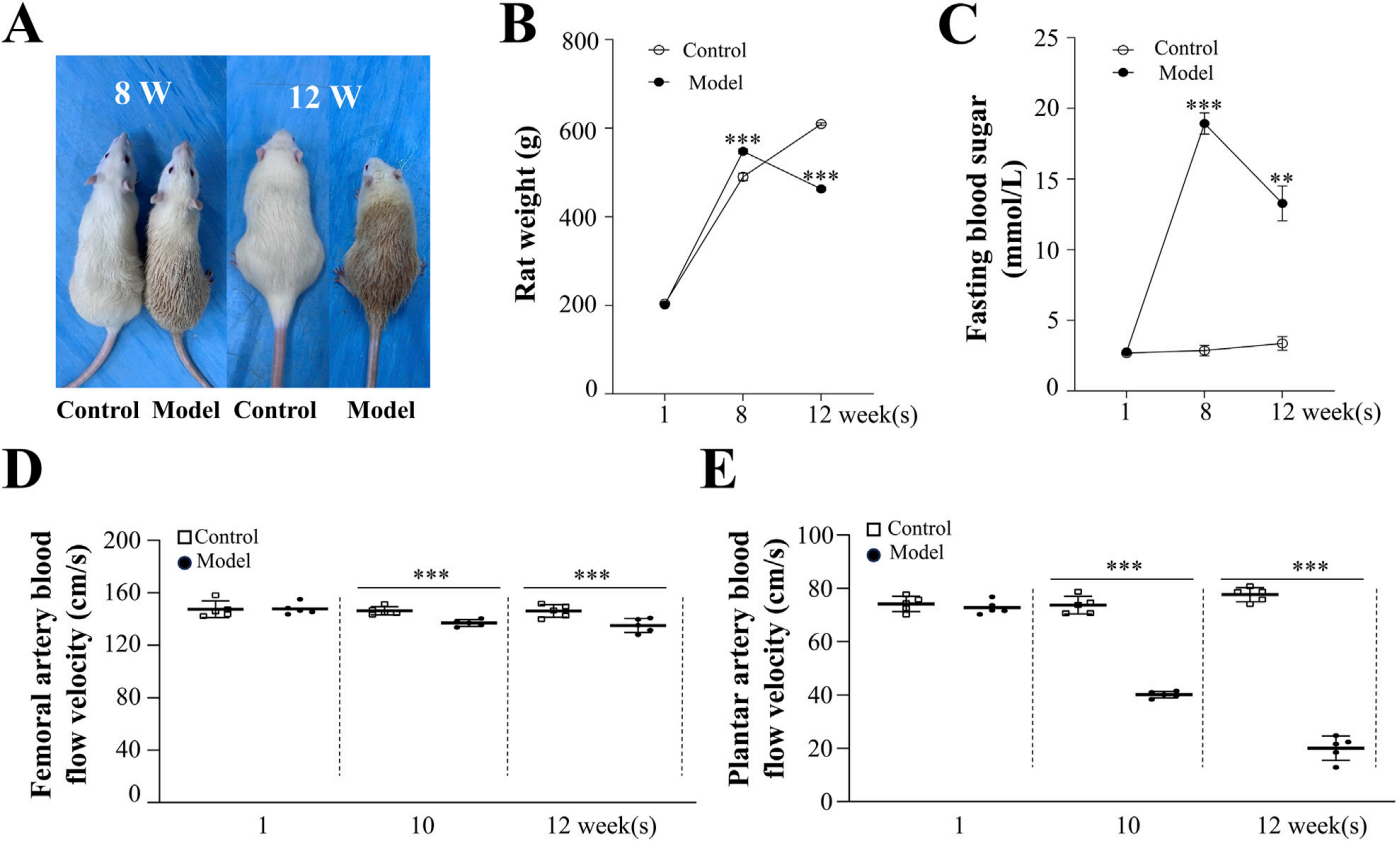
Morphological and Physiological Effects of High-Risk Diabetic Foot in Rats. **(A)** Morphological changes in control and model rats at weeks 8 and 12. Changes in body weight **(B)** and blood glucose levels **(C)** in control and model rats at weeks 1, 8, and 12. **(D)** Femoral artery blood flow velocity in rats. **(E)** Changes in plantar artery blood flow velocity in rats. Results are expressed as mean ± SEM. Compared to the control group: **p < 0.01, ***p < 0.001.

Rats in the model group exhibited weight loss following STZ intervention. As shown in Figure 1, blood glucose levels in the model group (13.28 ± 1.24 mmol/L, *p* < 0.001) were significantly higher than those in the control group (3.36 ± 0.48 mmol/L) and exceeded the critical value of 11.1 mmol/L at week 12, indicating successful establishment of the diabetes mellitus model (Wickramasinghe et al., 2022). To directly assess blood flow in the lower limbs of diabetic rats, LDF was employed to evaluate the femoral and plantar arteries. At week 12, the femoral artery blood flow velocity in the model group (135.02 ± 2.40 cm/s, *p* < 0.001) was lower compared to the control group (146.09 ± 2.15 cm/s). The plantar artery flow velocity showed an even greater reduction in the model group (19.99 ± 2.05 cm/s, *p* < 0.001) compared to the control group (77.63 ± 1.17 cm/s). These results indicate that significantly reduced blood flow in the lower limbs is a characteristic feature of diabetes mellitus.

### 3.2 FOE significantly improved plantar temperature and MNCV in foot

**FIGURE 2:**
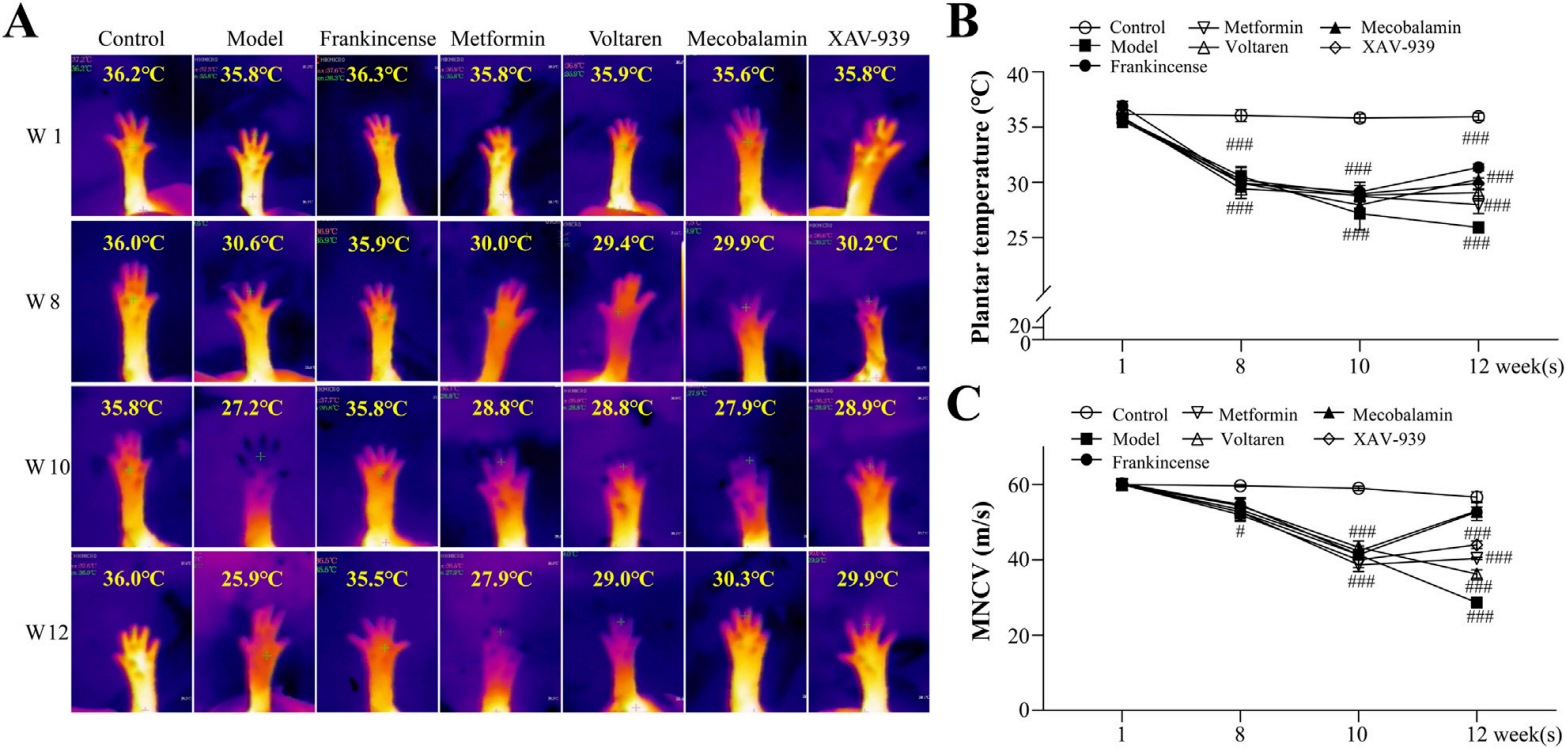
Effect of Frankincense Treatment on Plantar Temperature and MNCV in Rats with High-Risk Diabetic Foot. **(A)** Thermal imaging of the plantar region at weeks 1, 8, 10, and 12; plantar thermography images of rats from the negative control, model, FOE, metformin, voltaren, mecobalamin, and XAV-939 treatment groups. **(B)** Plantar temperature in rats. **(C)** MNCV in rats. Data are presented as mean ± SEM. Compared to the model group: #*p* < 0.05, ###*p* < 0.001.

Plantar temperature serves as an indicator of blood supply adequacy to distal capillaries and the smoothness of blood circulation (Rodrigues et al., 2022). As shown in Figure 2, at week 12, the plantar temperature of rats in the model group (25.9 °C ± 0.5 °C, *p* < 0.001) was significantly lower than that of the control group (35.9 °C ± 0.3 °C), highlighting significant microcirculation disorders and a reduction in local temperature caused by diabetes mellitus. Concurrently, rats in the model group exhibited a significantly lower MNCV (29.2 ± 0.5 m/s, *p* < 0.001) compared to the control group (57.4 ± 0.9 m/s, *p* < 0.001), indicating plantar sensory dullness as another characteristic feature of diabetes mellitus.

After 2 weeks of treatment, the plantar temperature of diabetic rats improved with various interventions. Metformin increased plantar temperature to 27.9 °C ± 0.8 °C (*p* > 0.05), while mecobalamin (30.3 °C ± 0.9 °C, *p* < 0.001), voltaren (31.3 °C ± 0.3 °C, *p* < 0.001), and the β-catenin inhibitor XAV-939 (29.1 °C ± 0.7 °C, *p* < 0.001) showed greater efficacy. FOE demonstrated the most notable improvement, raising the temperature to 31.3 °C ± 0.3 °C (*p* < 0.001). Similarly, MNCV increased to varying extents across treatments, with FOE (53.2 ± 0.9 m/s, *p* < 0.001) and mecobalamin (51.0 ± 1.9 m/s, *p* < 0.001) achieving the most pronounced effects, while metformin produced the smallest improvement (36.0 ± 0.7 m/s, *p* < 0.05). These findings indicate that FOE provides significant and stable improvements in both plantar temperature and MNCV.

### 3.3 FOE repaired microvascular damage and improved microcirculation disorders in the foot

**FIGURE 3:**
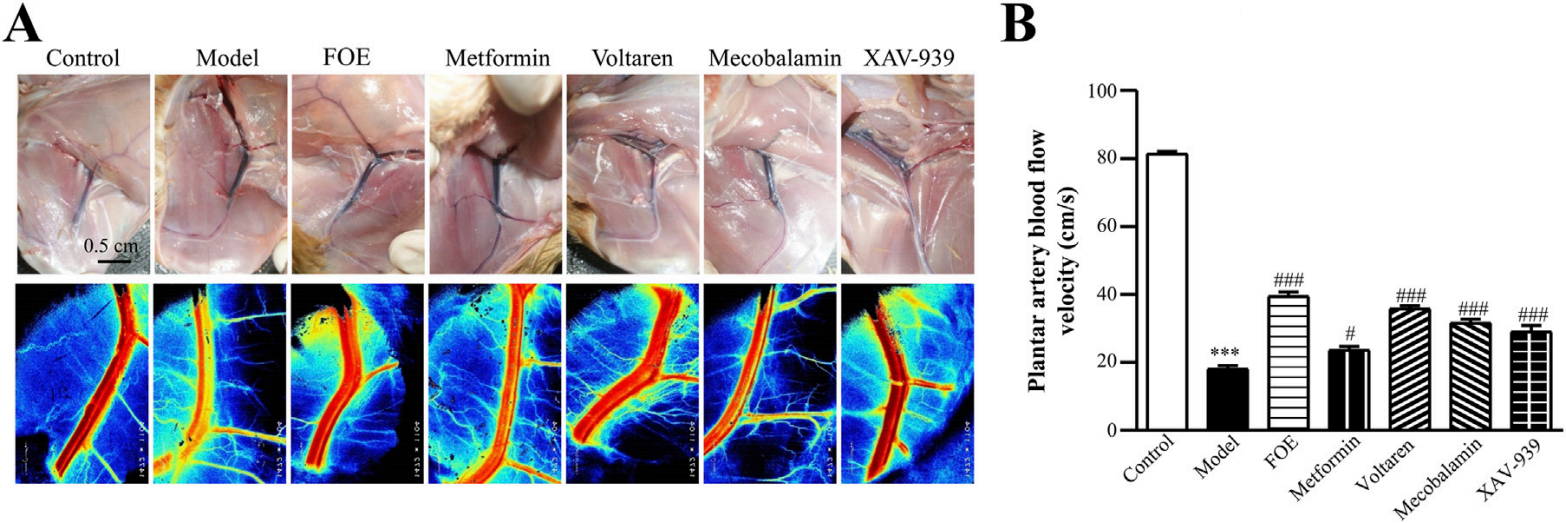
Effect of Frankincense Treatment on Femoral and Plantar Artery Blood Flow in Rats with High-Risk Diabetic Foot. **(A)** Laser Doppler flowmetry imaging of the femoral artery in rats at week 12. Scale bars: 0.5 cm. **(B)** Plantar artery blood flow velocity in rats. Data are presented as mean ± SEM. Compared to the control group: ***p < 0.001. Compared to the model group: #*p* < 0.05, ###*p* < 0.001.

Direct observation of rat femoral arteries (Figure 3) revealed that the femoral arteries of healthy rats appeared bright red with a uniform lumen at week 12. In contrast, the femoral arteries of diabetic rats displayed a purplish-red hue, along with arterial dilatation, suggesting varying degrees of blockage. Additionally, an increase in the density of microvascular branches surrounding the femoral artery was noted. Drug treatments improved plantar artery blood flow velocity in all groups. Metformin (23.6 ± 1.1 cm/s, *p* < 0.05), mecobalamin (31.6 ± 1.1 cm/s, *p* < 0.001), voltaren (35.8 ± 0.9 cm/s, *p* < 0.001), and XAV-939 (29.0 ± 1.9 cm/s, *p* < 0.001) all demonstrated efficacy, with FOE showing the greatest improvement (39.4 ± 1.4 cm/s, *p* < 0.001). These results suggest that the improved plantar temperature is attributable to the restoration of plantar vascular circulation by FOE.

**FIGURE 4:**
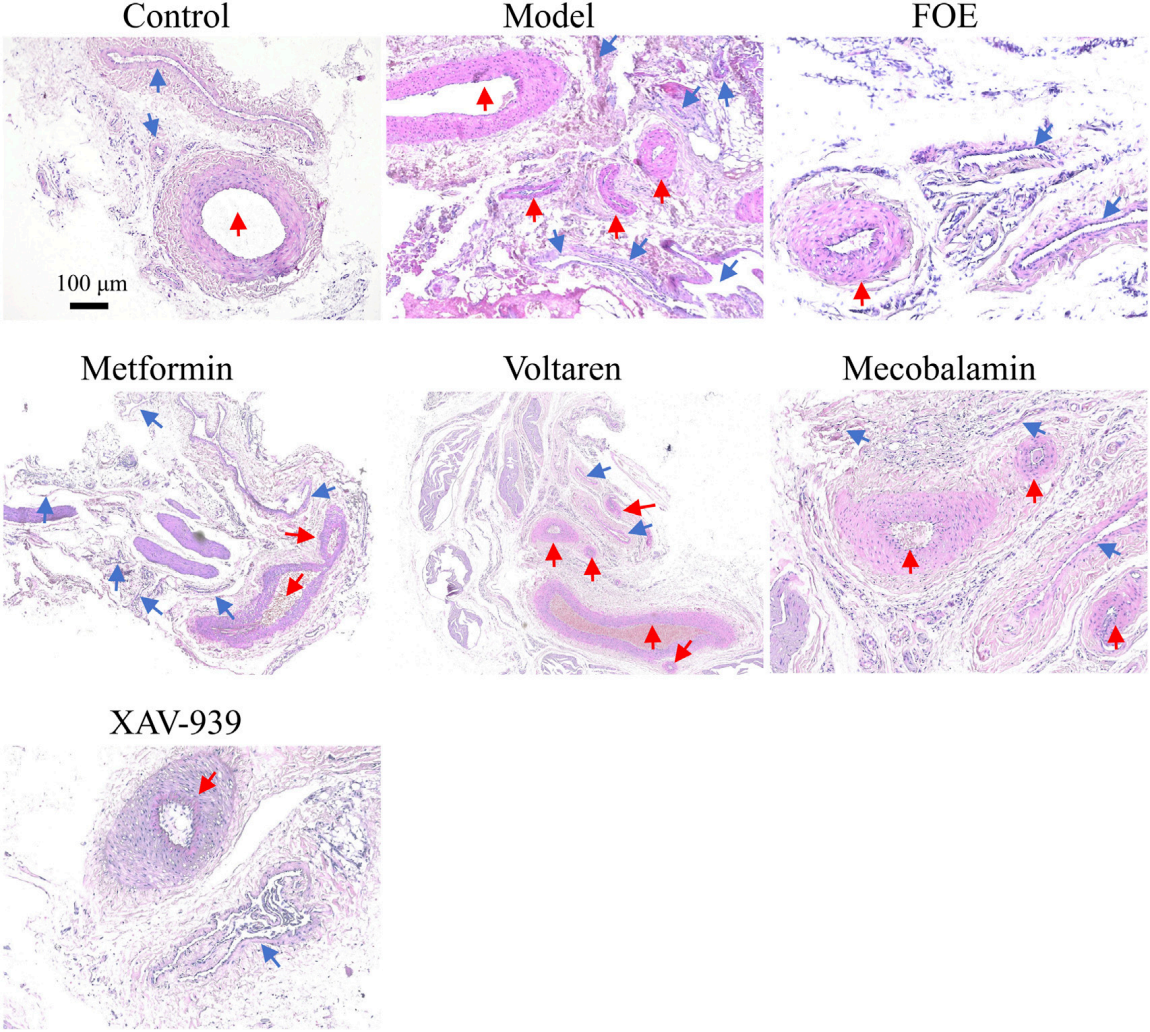
Effect of Frankincense treatment on peripheral vessels of the Femoral Arteries Representative images of histopathological changes in the arteriovenous plexus surrounding the femoral arteries of the rat limb. Scale bars: 100 µm for the top images, 50 µm for the bottom images. Blue arrows indicate venous vessels, and red arrows indicate arterial vessels.

AV-shunts allow blood to bypass capillaries by flowing directly from small arteries into small veins, leading to microvascular atrophy due to inadequate blood supply (Walløe, 2016). To examine the impact of diabetes mellitus on neovascularization and bifurcation in the lower limbs, we analyzed tissues surrounding the femoral arteries of rats (Figure 4). Pathological analysis showed that compared to healthy rats, the arterial and venous vessel walls in the model group were thickened and structurally disorganized, with irregular intimal hyperplasia and uneven lumen thickness. Additionally, more microvessels were observed around the arteriovenous plexus in diabetic rats, likely due to AV-shunt formation caused by diabetes-induced microcirculatory disorders, which stimulated neovascularization. After treatments with metformin, voltaren, and mecobalamin, the arteries and veins retained some micro-arteries and micro-veins. However, FOE and XAV-939 treatments reduced the number of microvessels. Following FOE treatment, the arteries exhibited a well-defined structure, whereas veins in the XAV-939 group continued to display a high number of bifurcations.

**FIGURE 5:**
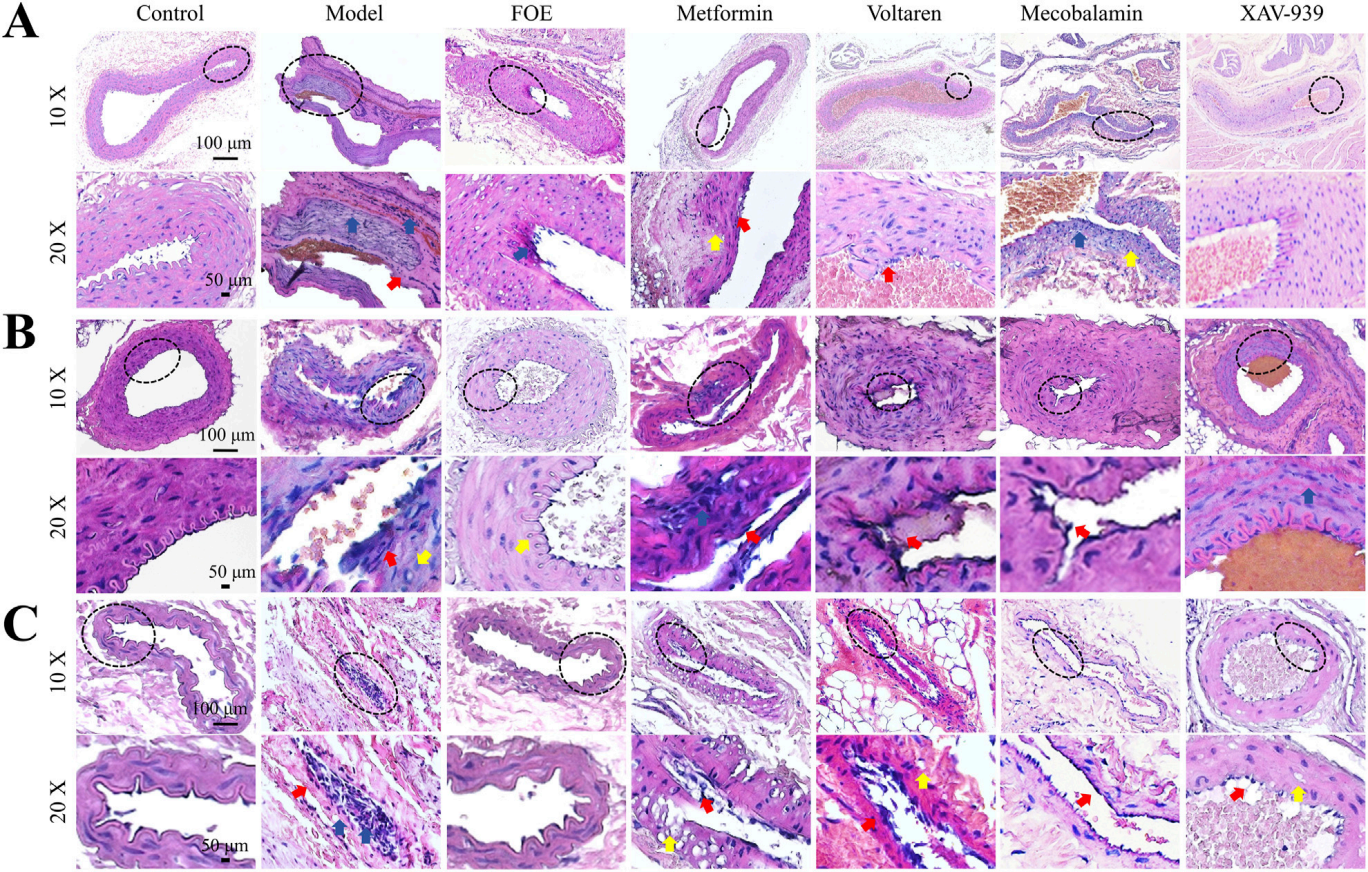
Effect of Frankincense Treatment on Histopathological Changes in Foot Blood Vessels of Rats Histopathological changes in the femoral arteries **(A)**, plantar arteries **(B)**, and microvessels **(C)** of rats with high-risk diabetic foot. Scale bars: 100 µm. Red arrows indicate intimal damage, yellow arrows indicate vacuole formation, and blue arrows indicate inflammatory infiltration in blood vessels.

As shown in Figure 5, the femoral arteries, plantar arteries, and plantar capillaries of diabetic rats exhibited varying degrees of damage, including rupture of the vascular intima, loose fibrous connections in small vessels, and, in some cases, separation accompanied by small vacuoles. These lesions resulted in structural damage and functional decline of the endothelial layer in small and medium-sized vessels, further highlighting the detrimental effects of diabetes on the blood vessels of the lower extremities. However, after FOE treatment, the plantar arteries and capillaries displayed intact and continuous tunica intima with a uniform wave-like structure. There was a reduction in inflammatory cells within the vessels, tight connections among endothelial cells, and an increase in vessel density. These observations suggest that FOE can effectively repair vascular damage in the foot.

**FIGURE 6:**
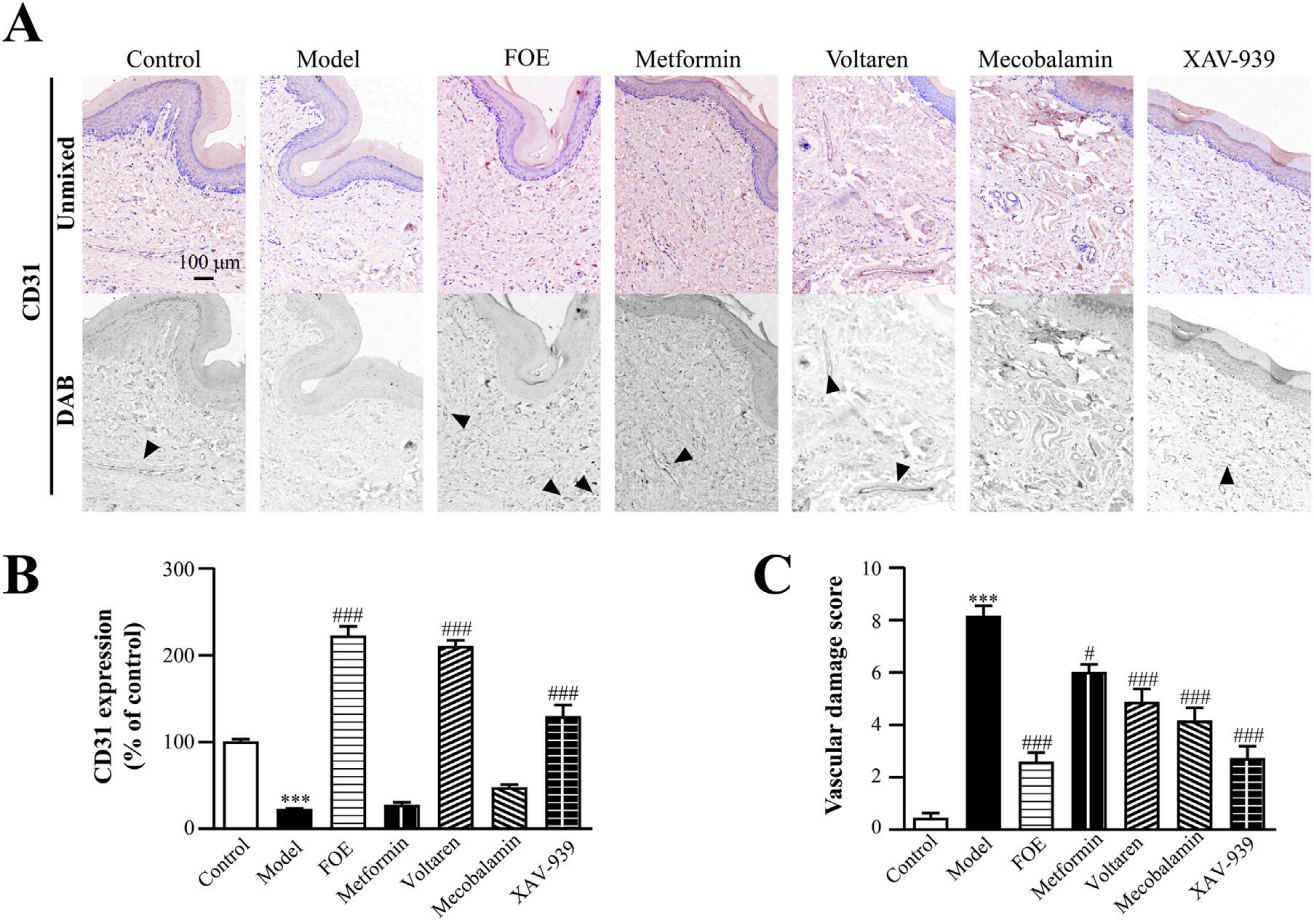
Effect of Frankincense Treatment on the Plantar Skin of Rats with High-Risk Diabetic Foot. **(A)** Representative photomicrographs of CD31 staining in the plantar surface of rats. Black arrows indicate the location of target protein expression. **(B)** Quantitative analysis of immunohistochemical staining for CD31 protein. **(C)** Vascular damage scores in the plantar surface of rats. Scale bars: 100 µm. Results are expressed as mean ± SEM. Compared to the control group: ***p < 0.001. Compared to the model group: #*p* < 0.05, ###*p* < 0.001.

To better understand vascular neovascularization in the distal limbs of the rats, CD31 staining was performed on plantar tissues. Interestingly, the findings contrasted with the results observed in tissues surrounding the femoral artery. As shown in Figure 6, CD31 protein expression in the model group was significantly down-regulated (22% ± 1% of the control; *p* < 0.001) compared to the control group (100% ± 3%), indicating atrophy or disintegration of plantar microvessels and a significant reduction in vascular density in diabetic rats. This result demonstrates that proximal AV-shunts cause inadequate blood supply to the distal extremities, ultimately impairing vascular structure and density in distal tissues. Following treatments with voltaren (210% ± 7% of the control; *p* < 0.001) and XAV-939 (129% ± 14% of the control; *p* < 0.001), CD31 expression significantly increased, with the FOE group showing the highest upregulation (222% ± 11% of the control; *p* < 0.001). These findings indicate that FOE may repair vascular endothelial damage effectively. In contrast, metformin (26.8% ± 4% of the control; *p* > 0.05) and mecobalamin (47% ± 5% of the control; *p* > 0.05) did not significantly upregulate CD31 expression.

By evaluating vascular damage scores based on pathological changes in blood vessels and CD31 expression, it was evident that the diabetic condition significantly worsened vascular damage. FOE (2.6 ± 0.4, *p* < 0.001) and XAV-939 (2.7 ± 0.5, *p* < 0.001) demonstrated the most notable effects on repairing plantar microvascular damage.

These findings suggest that for the proximal arteriovenous plexus, FOE reduces the formation of microvessels surrounding the femoral artery by inhibiting the development of AV-shunts. This reduction minimizes blood shunting and restores normal blood flow to the foot. Regarding foot microcirculation, FOE appears to support vascular homeostasis and promote neovascularization by reversing the downregulation of CD31. This process increases the density of microvessels in the plantar region, ensuring smooth blood circulation and contributing to the normalization of foot temperature in diabetic rats.

### 3.4 FOE repaired neuropathy by regulating the low expression of TRPV3

Long-term vasculopathy associated with diabetes adversely impacts nerve function. TRPV3, expressed in sensory nerve cells and skin, plays a critical role in heat perception and pain sensation (Su et al., 2023). To evaluate nerve sensitivity, TRPV3 expression was used as an indicator of neurological function.

**FIGURE 7:**
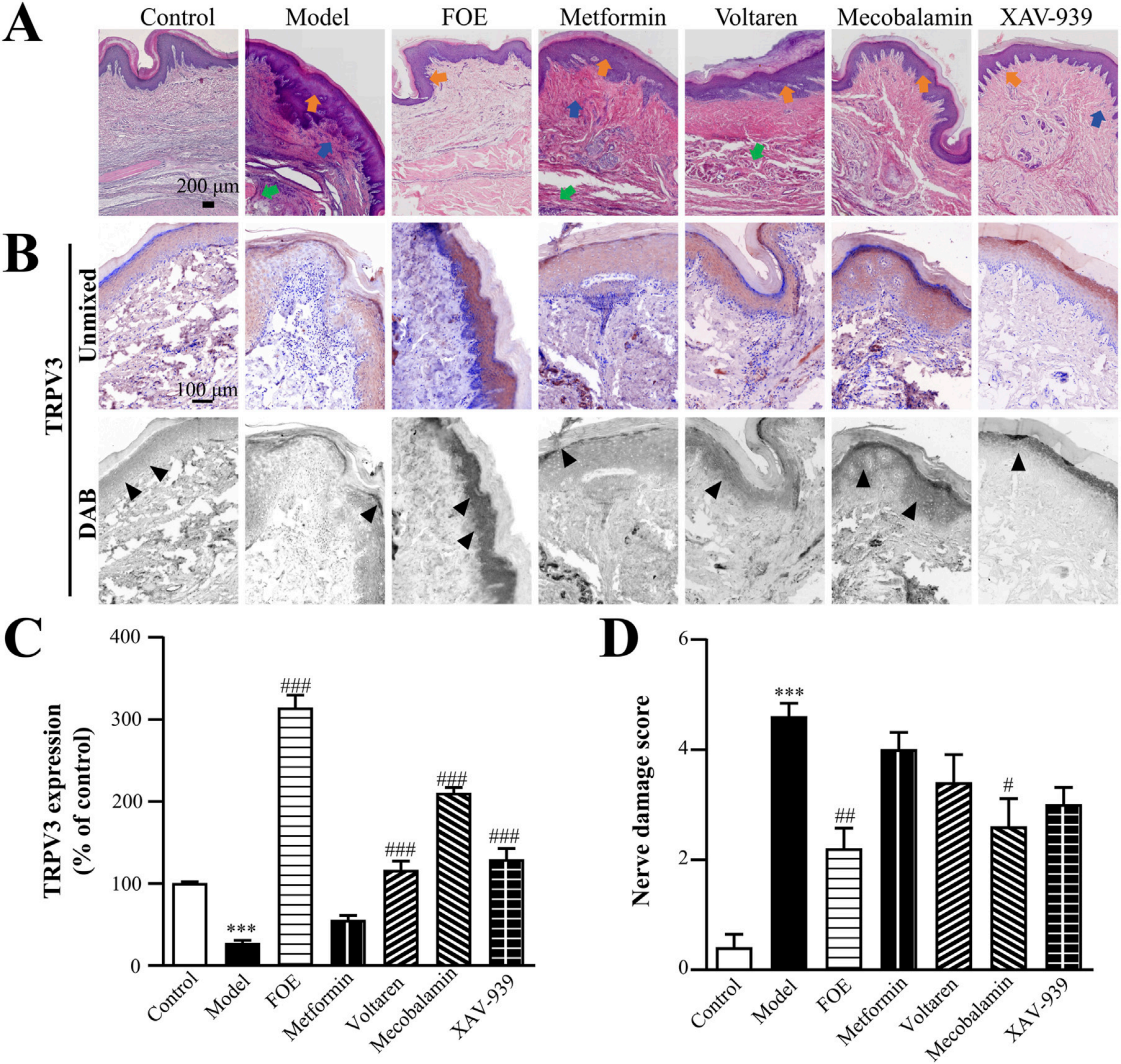
Effect of Frankincense Treatment on Nerve Injury in Rats with High-Risk Diabetic Foot. **(A)** Histopathological changes in the plantar tissue of rats. Orange, blue, and green arrows indicate abnormalities in the spinous layer, inflammatory infiltration, and atrophy of the muscularis propria in rat plantar skin tissue, respectively. Scale bars: 200 µm. **(B)** Representative images of immunohistochemical staining for TRPV3 in the plantar skin of rats. Black arrows indicate the location of target protein expression. Scale bars: 100 µm. **(C)** Quantitative multispectral imaging of TRPV3 protein. **(D)** Nerve damage scores in rats. Results are expressed as mean ± SEM. Compared to the control group: ***p < 0.001. Compared to the model group: #*p* < 0.05, ##*p* < 0.01, ###*p* < 0.001.

The results (Figure 7) showed that the plantar skin of the control group was structurally intact, with TRPV3 localized in the epidermis, particularly in the spinous layer. In contrast, the model group exhibited a thinner epidermis, abnormal spinous layers, and significantly down-regulated TRPV3 expression (27% ± 4% of control, *p* < 0.001) compared to the control group (100% ± 2%). Furthermore, the density of micro vessels and nerve endings was significantly reduced. These findings suggest that chronic diabetes damages nerve endings, impairs the sensory function of the plantar foot, and diminishes the ability of plantar nerves to regulate the state of microvessels. Consequently, this weakens the self-repairing capacity of the plantar skin barrier, making the foot more susceptible to small wounds under stimulation.

Following treatment, the plantar skin structure of the drug-administered groups remained intact, with normal dermal thickness, regular spinous layer projections, increased density of microvessels and nerve endings, and a reduction in inflammatory cells in the basal epithelial and muscular layers. TRPV3 expression levels improved in the metformin group (55% ± 6% of control, *p* > 0.05), mecobalamin group (210% ± 7% of control, *p* < 0.001), voltaren group (116% ± 11% of control, *p* < 0.001), and XAV-939 group (129% ± 14% of control, *p* < 0.001). However, FOE demonstrated the most significant improvement in TRPV3 expression (314% ± 16% of control, *p* < 0.001).

Nerve damage scores, based on plantar pathological changes and TRPV3 expression (Figure 7D), further supported these findings. Compared to the control group (0.4 ± 0.2), the model group showed significantly higher nerve injury scores (4.6 ± 0.2, *p* < 0.001). Among the treatment groups, FOE (2.2 ± 0.4, *p* < 0.001) and mecobalamin (2.6 ± 0.5, *p* < 0.05) demonstrated the most pronounced therapeutic effects on plantar neuropathy. These results confirm that FOE can effectively repair terminal nerve damage and improve nerve function.

### 3.5 FOE inhibited persistent inflammation in high-risk diabetic foot

**FIGURE 8:**
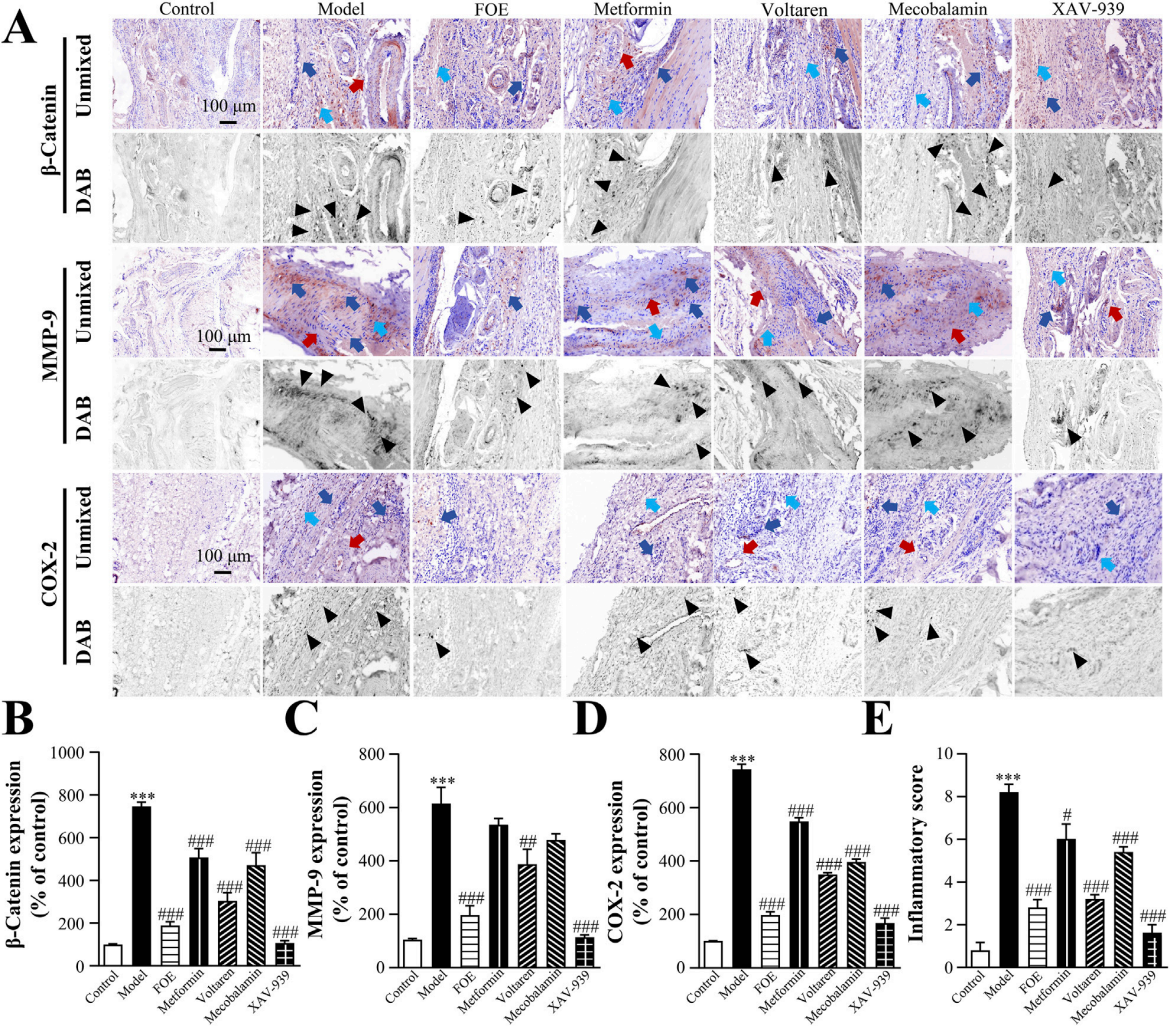
Effect of Frankincense Treatment on Inflammatory Factors in Rats with High-Risk Diabetic Foot. **(A)** Representative images of immunohistochemical staining for β-catenin, MMP-9, and COX-2 proteins in the plantar tissue of rats. Scale bars: 100 µm. Black arrows indicate the location of target protein expression. Red, blue, and light blue arrows indicate vascular wall thickening, inflammatory infiltration, and collagen disorders in rat plantar tissue, respectively. Quantitative multispectral imaging of β-catenin **(B)**, MMP-9 **(C)**, and COX-2 **(D)** proteins in the plantar tissue of rats. **(E)** Inflammatory scores in the plantar tissue of rats. Results are expressed as mean ± SEM. Compared to the control group: ***p < 0.001. Compared to the model group: #*p* < 0.05, ##*p* < 0.01, ###*p* < 0.001.

Vascular and nerve damage contribute to the accumulation of inflammation in the foot, with persistent inflammation playing a critical role in the progression of high-risk diabetic foot. The plantar tissues of diabetic rats showed abnormally high expression levels of β-catenin, MMP-9, and COX-2 (Figure 8), indicating a robust inflammatory response. Diabetic rats, subjected to impaired foot circulation caused by ice walking, combined with vasculopathy, neuropathy, and inflammation in the lower limbs, were successfully modeled as high-risk diabetic foot rats.

Following treatment, β-catenin expression was significantly reduced in the XAV-939 group (106% ± 12% of control, *p* < 0.001) and the voltaren group (302% ± 40% of control, *p* < 0.001), compared to the model group (746% ± 21% of control). XAV-939 also effectively inhibited COX-2 (197% ± 19% of control, *p* < 0.001) and MMP-9 (114% ± 9% of control, *p* < 0.001), suggesting that COX-2 and MMP-9 may be regulated by β-catenin in the inflammatory response. FOE demonstrated the strongest anti-inflammatory effects, significantly down-regulating β-catenin (188% ± 18% of control, *p* < 0.001), MMP-9 (196% ± 72% of control, *p* < 0.001), and COX-2 (196% ± 17% of control, *p* < 0.001).

Based on the expression levels of inflammation-related factors, a comprehensive assessment of the degree of inflammatory infiltration was conducted (Figure 8E). The model group exhibited a significantly higher inflammatory score (8.2 ± 0.5, *p* < 0.001) compared to the control group (0.8 ± 0.5). FOE showed the most pronounced inhibitory effects on persistent plantar inflammation, reducing the inflammatory score to 2.8 ± 0.5 (*p* < 0.001). These findings confirm that FOE effectively inhibits persistent inflammation in high-risk diabetic foot by down-regulating the expression of inflammatory factors β-catenin, MMP-9, and COX-2.

## 4 Discussion

The decrease in plantar temperature observed in this study reflects diabetes-induced microcirculatory disturbances and reduced blood flow, primarily due to thickened and less elastic blood vessel walls caused by chronic hyperglycemia. These changes impair the ability of blood to flow to the feet (Fan et al., 2020). Additionally, diabetic rats in this experiment exhibited slowed MNCV, which is likely associated with nerve damage and energy deficiency (Carrington et al., 2002).

A combination of endothelial dysfunction, inflammation, and nerve damage may contribute to the development of DFU (Rismayanti et al., 2022). Our findings revealed the pathological changes experienced by plantar arteries and foot capillaries in hyperglycemic states, including endothelial cell damage, disruption of the endothelial layer structure, and microcirculatory disturbances (Rask-Madsen and King, 2013). These abnormalities not only compromise vascular structure and function but also lead to ischemia and metabolic irregularities in local tissues (Mizisin and Weerasuriya, 2011). Furthermore, we observed that the formation of AV-shunts hindered normal blood flow to the extremities, particularly the soles of the feet. This resulted in micro-arteriolar atrophy due to inadequate blood supply (Taylor et al., 2024). Pathological analyses of the vessels surrounding and distal to the AV-shunts demonstrated significant vascular dilation, vessel wall thickening, and endothelial irregularities, all indicative of severe vasculopathy. The reduced expression of CD31 at the extremities, along with blurring of the vascular structure and significant decreases in vascular density, highlighted the detrimental effects of AV-shunts on foot microcirculation. These effects directly contributed to decreased plantar temperature and slowed blood flow in the plantar arteries of diabetic rats.

We observed a clear correlation between plantar nerve ending injury, reduced blood vessel density, and muscle atrophy in diabetic rats. These findings highlight an interconnected pathological process in which reduced blood vessel density leads to an inadequate blood supply to the plantar region. This insufficiency adversely affects the health and function of nerve endings and muscle tissue (Imamura et al., 2022). The damaged nerve endings were unable to conduct signals efficiently, resulting in reduced plantar sensory function, as evidenced by the low MNCV in rats. Additionally, impaired nerve endings diminished protective sensation in the foot, increasing the risk of foot injuries and subsequent ulcer formation (Feldman et al., 2019). In the plantar skin of diabetic rats, TRPV3 expression was significantly down-regulated, which may imply a reduced ability to sense temperature or pain (Su et al., 2023). TRPV3 also plays a role in regulating skin barrier function, and its abnormal expression can impair the skin’s self-repairing ability or make it hypersensitive (Tóth et al., 2014). This reflects the extensive impact of diabetes on the nervous system, particularly in the pathology of high-risk diabetic foot, where nerve damage exacerbates the risk to foot health (Kim et al., 2012).

Persistent inflammation further compounds tissue damage and exacerbates diabetic foot injuries (Tsalamandris et al., 2019). The downstream inflammatory factor MMP-9, a product of the Wnt/β-catenin signaling pathway, contributes to the degradation of collagen and other matrix proteins in damaged tissues (Xiao et al., 2022), Abnormal expression and activation of MMP-9 can disrupt normal tissue integrity and accelerate ulcer formation (Chang and Nguyen, 2021). Elevated MMP-9 levels are commonly observed in patients with diabetic foot and are associated with impaired tissue repair, ulcer formation, and disease progression (Liu et al., 2009). MMP-9 elevation may promote vascular endothelial cell damage and degradation of vascular wall proteins (Kato et al., 2014), worsening the progression of diabetic foot. The transition from high-risk diabetic foot to DFU is partially driven by unstable blood glucose levels and diabetes-associated complications such as microangiopathy. These factors can lead to an abnormal increase in MMP-9 expression and activity, thereby inducing the formation and progression of DFU.

We found that neither commonly used clinical hypoglycemic drugs nor treatments targeting a single symptom were effective in managing high-risk diabetic foot. In contrast, frankincense demonstrated significant efficacy in treating this condition. In addition to its known ability to improve microcirculation in frostbitten skin (Zhang et al., 2025), frankincense showed remarkable therapeutic effects on high-risk diabetic foot. Firstly, frankincense increased the density of blood vessels in the foot and restored microcirculation by upregulating CD31 expression and preventing the formation of AV-shunts. Secondly, after normalizing microvascular function, frankincense reduced nerve damage and repaired the skin barrier by upregulating TRPV3 expression. With the restoration of vascular and nerve integrity, the inflammatory response was subsequently diminished. Ultimately, frankincense exerted anti-inflammatory effects by inhibiting the β-catenin/COX-2-MMP-9 inflammatory pathway, thereby achieving the goal of preventing and treating high-risk diabetic foot.

In summary, this study established the close relationship between the pathological changes in high-risk diabetic foot and microcirculation disturbances caused by prolonged hyperglycemia. Frankincense effectively improved microcirculation disorders in the foot, protected nerves, reduced inflammation, and regulated the expression of CD31, TRPV3, and key inflammation-related factors (β-catenin, MMP-9, and COX-2). These findings provide strong scientific support for the application of frankincense in the treatment and early intervention of high-risk diabetic foot.

## Data Availability

The original contributions presented in the study are included in the article/Supplementary Material, further inquiries can be directed to the corresponding authors.
