# Supplementary material for: Topical frankincense treatment on relieving high-risk diabetic foot in rats by reducing inflammation and improving microcirculation
Source: Front Pharmacol. 2025 Sep 16;16:1564236. doi: 10.3389/fphar.2025.1564236 (PMC12481894; doi:10.3389/fphar.2025.1564236)
Supplement: Supplementary file 1 [file Supplementaryfile2.doc]

**Supplementary Material**

Topical frankincense treatment on relieving high-risk diabetic foot in rats by reducing inflammation and improving microcirculation

**Si-Yuan Ma^1, †^, Xin Yin^1,2, †^, Zhao Zhang^1,3^, Yu-Sang Li^1, *^, He-Bin Tang^1, *^**

* Corresponding authors:

Yu-Sang Li: [liys2006@mail.scuec.edu.cn, tel/0086-27-67841196;](mailto:liys2006@mail.scuec.edu.cn, tel/0086-27-67841196;)

He-Bin Tang: [hbtang2006@mail.scuec.edu.cn](mailto:hbtang2006@mail.scuec.edu.cn,tel/0086-27-67842332),[tel/0086-27-67842332](mailto:hbtang2006@mail.scuec.edu.cn,tel/0086-27-67842332);

**GC-MS analysis of FOE**

The chemical components of Frankincense oil extracts (FOE) were detected by GC-MS on a Thermo Scientiﬁc TRACE 1300 GC Ultra system equipped with a TR-35MS capillary column (30 m×0.25 mm×0.25 μm) coupled to an ISQ system in electron ionization mode. The initial oven temperature was 50°C. Samples were maintained at 50 °C for 1 min, and the temperature was increased at a rate of 50°C/min to 200°C, where it was held for 2 min. The injector and transfer line temperatures were both 290°C.The injection volume was 1 μL. Ions were generated by a 70 eV electron beam at an ionization current of 50 μA and an ion source temperature of 250°C. The mass spectra were recorded in full scan mode (m/z 50-500) for qualitative analysis. All analysis was subjected to strict quality assurance and control procedures, including the analysis of procedural ﬁeld blanks and standard samples for validation of analytical methodology. The contents of α-pinene,linalool, and 1-octanol were calculated by external standard method.


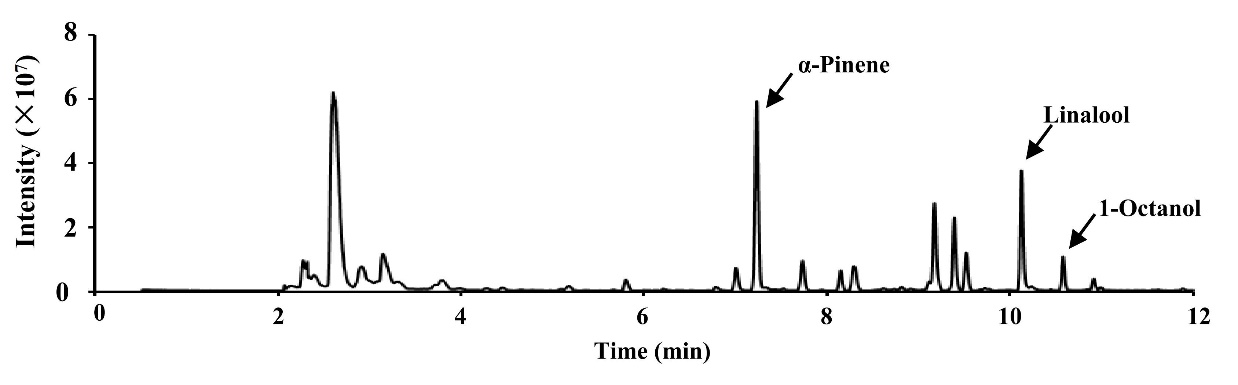


**Supplementary Figure 1. GC-MS analysis of the components of Frankincense oil extracts (The relevant data has already been published in the Journal of Ethnopharmacology. 2016,179: 22-26).**
