# Supplementary material for: Topical frankincense treatment on relieving high-risk diabetic foot in rats by reducing inflammation and improving microcirculation
Source: Front Pharmacol. 2025 Sep 16;16:1564236. doi: 10.3389/fphar.2025.1564236 (PMC12481894; doi:10.3389/fphar.2025.1564236)
Supplement: Supplementary file 2 [file Supplementaryfile1.docx]

Supplementary Material

# Supplementary Tables

Scoring criteria for vascular damage, nerve damage, and inflammation were established based on histopathological findings and the expression levels of related proteins.

## Supplementary Table 1. Vascular injury scoring scale of rats

| **Scoring Item** | **0 Point** | **1 Point** | **2 Points** | **3 Points** |
| --- | --- | --- | --- | --- |
| **Local Thickening of the Arterial Wall** | No apparent thickening | Mild thickening without structural distortion | Moderate thickening with some loss of structural integrity | Severe thickening with significant loss of structural integrity |
| **Homogeneous, Transparent, Structureless Changes in the Arterial Media (Glassy Changes)** | Absent | Present in a small area | Present in a moderate area | Extensive throughout the arterial media |
| **Integrity of the Small to Medium Vessel Endothelium** | Intact continuous endothelium with a uniform，wavy structure | Minor damage or breakage, slight discontinuity | Moderate damage with significant discontinuity and some areas of separation | Severe damage with widespread discontinuity, separation, and formation of vacuoles |
| **Integrity of the Small to Medium Vessel Endothelium** | No significant inflammation or fibrosis | Mild inflammation with minimal fibrosis | Moderate inflammation with evident fibrosis | Severe inflammation with extensive fibrosis |
| **Vascular Remodeling near AV-Shunt** | No evidence of remodeling | Slight vascular dilation and mild wall thickening | Moderate dilation and thickening with irregular endothelial proliferation | Severe vascular dilation, wall thickening, and disorganized structure |

## Supplementary Table 2. Nerve injury injury scoring scale of rats

| **Scoring Item** | **0 Point** | **1 Point** | **2 Points** | **3 Points** |
| --- | --- | --- | --- | --- |
| **Nerve Ending Density** | No noticeable changes | Slight deformation or breakage of nerve fibers, less infiltration of inflammatory cells | Obvious nerve fiber breakage, deformation, moderate infiltration of inflammatory cells | Significant decrease in never ending density, severe infiltration of inflammatory cells or fibrosis, local hemorrhagic foci |
| **plantar cortex Structural Changes** | Cortical structure intact, normal thickness | Slight thinning of the cortical layer, minor abnormalities in the acanthosis | Significant thinning of the cortical layer, acanthosis abnormalities or disappearance, reduction in nerve endings and micro-vessel density | Severe destruction of the cortical structure, disappearance of acanthosis, significant reduction in nerve endings and micro-vessel density, muscle layer atrophy |

## Supplementary Table 3. Inflammation scoring scale of rats

| **Scoring Item** | **0 Point** | **1 Point** | **2 Points** | **3 Points** |
| --- | --- | --- | --- | --- |
| **Inflammatory Cell Infiltration Density** | No apparent inflammatory cell infiltration | Mild infiltration (visible few lymphocytes, macrophages, and neutrophils) | Moderate infiltration (clear accumulation of inflammatory cells but not forming a large inflammatory reaction area) | Severe infiltration (widespread accumulation of inflammatory cells, forming a clear inflammatory reaction area) |
| **Local Tissue Structure Changes** | No apparent changes | Slight enlargement of intercellular gaps, minor disorganization of collagen fibers | Clear enlargement of intercellular gaps, disorganized arrangement of collagen fibers, swelling of a few cells | Extensive enlargement of intercellular gaps, severe disorganization of collagen fibers, swelling, and necrosis of many cells |
| **Inflammation Around Blood Vessels and Vascular Wall Thickness** | No infiltration of inflammatory cells, normal vascular wall thickness | Mild infiltration of inflammatory cells, slight thickening of the vascular wall | Moderate infiltration of inflammatory cells, clear thickening of the vascular wall | Severe infiltration of inflammatory cells, extensive thickening of the vascular wall |
| **Impact of Cytokines and Chemical Signals (if quantifiable)** | No increase | Slight increase | Significant increase | Extreme increase |
